# Acid sensing to inflammaging: mechanisms and therapeutic promise of GPR68 (OGR1) in aging-related diseases

**DOI:** 10.3389/fragi.2025.1684450

**Published:** 2025-10-20

**Authors:** Jianlei Wei, Fengxin Cui, Zhihua Huang, Zeping Li, Zebin Mao, Pengxia Zhang

**Affiliations:** ^1^ School of Basic Medicine, Jiamusi University, Jiamusi, Heilongjiang, China; ^2^ Department of Biochemistry and Molecular Biology, School of Basic Medical Sciences, Peking University, Beijing, China; ^3^ The Seventh People’s Hospital affiliated to Shanghai University of Traditional Chinese Medicine, Shanghai, China

**Keywords:** GPR68, chronic inflammation, immune response, aging-related diseases, therapeutic target

## Abstract

GPR68 is a proton-sensing G protein-coupled receptor with an activation threshold at extracellular pH values between 6.5 and 7.0. It is widely expressed in diverse cell types, particularly in fibroblasts and cancer cells, within inflammatory and tumor microenvironments. In inflammatory bowel disease patients, GPR68 expression is also significantly increased in macrophages and monocytes. GPR68 primarily modulates inflammatory responses through the Gq/11–phospholipase C–inositol 1,4,5-trisphosphate/Ca^2+^ signaling axis. Extracellular acidification first promotes GPR68 coupling with Gq/11, subsequently enhancing phospholipase Cβ activity and increasing IP_3_ production; IP_3_ then mediates Ca^2+^ release from the endoplasmic reticulum, activating calmodulin-dependent kinase and calcineurin, ultimately inducing NF-κB and NFAT nuclear translocation to upregulate inflammatory mediators such as IL-6, TNF-α, and COX-2. This cascade activates inflammatory signaling pathways, thereby driving cellular and tissue senescence and creating favorable conditions for the progression of age-related diseases. However, its long-term causal relationship requires further validation through prospective studies. Abnormal GPR68 expression is closely associated with chronic inflammation, acidosis, and fibrosis in diseases including osteoarthritis, atherosclerosis, chronic kidney disease, Alzheimer’s disease, Parkinson’s disease, glioblastoma (GBM), and pancreatic cancer. In GBM, knocking down GPR68 or using the GPR68 inhibitor ogremorphin significantly reduces tumor cell survival. Despite its potential as a therapeutic target, challenges remain, such as the unresolved crystal structure, the lack of *in vivo* causality, cell-type specificity, and context-dependent signaling mechanisms. Targeting GPR68 may offer novel therapeutic strategies for these pathological processes.

## 1 Introduction

**FIGURE 1:**
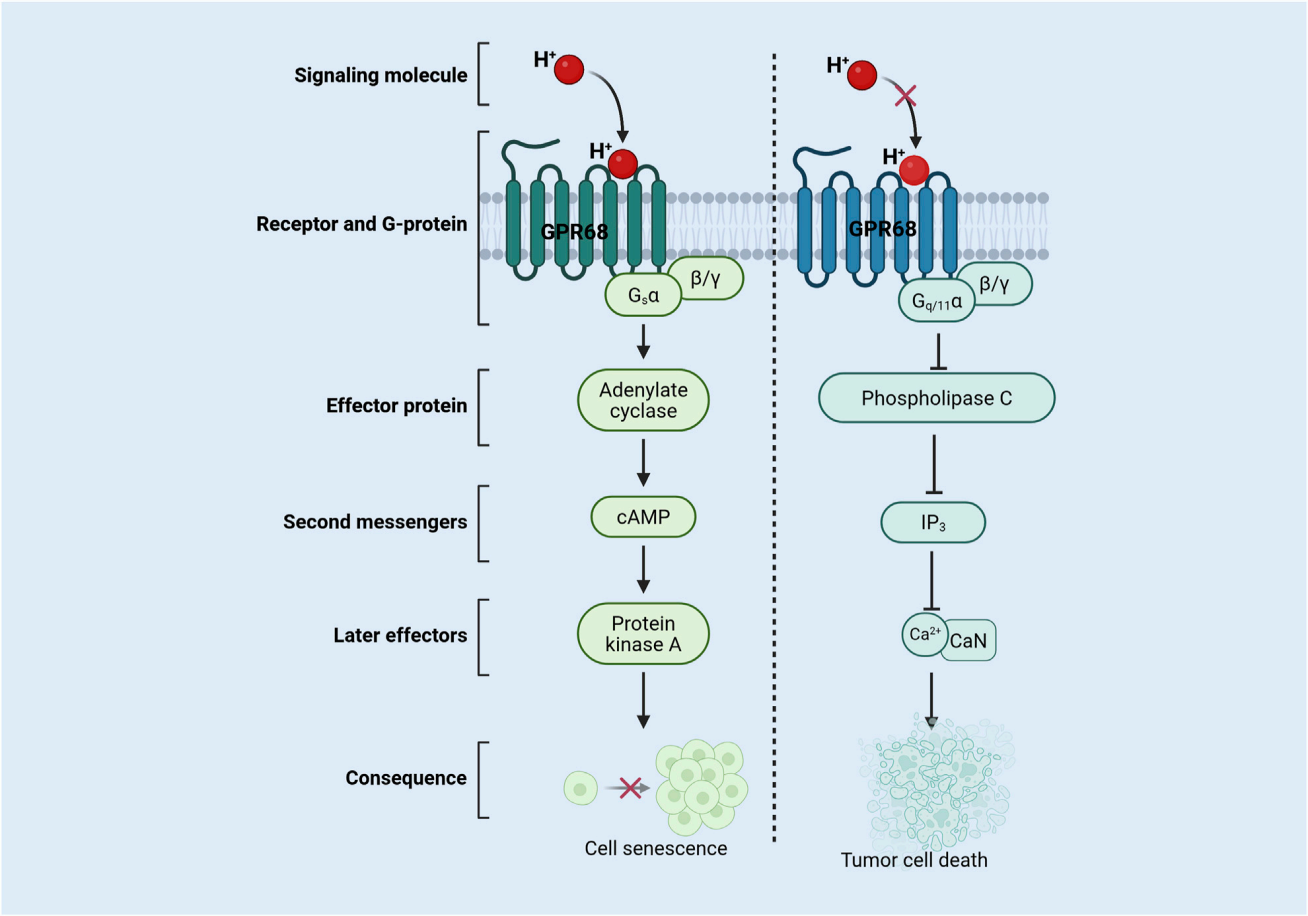
Mechanism of action of GPR68 in senescent cells and tumor cells. During aging, extracellular acidification activates the GPR68 signaling pathway, leading to restricted cell proliferation and inducing cellular senescence. In tumors, reduced extracellular acidification inhibits GPR68 pathway activation, thereby inducing tumor cell death. Abbreviations: CaN, calcineurin; cAMP, cyclic adenosine monophosphate; Gq/11, G protein alpha-q/11 subunit; Gsα, protein alpha-s subunit; H^+^, hydrogen ion; GPR68, G protein-coupled receptor 68; IP_3_, inositol 1,4,5-trisphosphate; β/γ, beta/Gamma Subunit.

**FIGURE 2:**
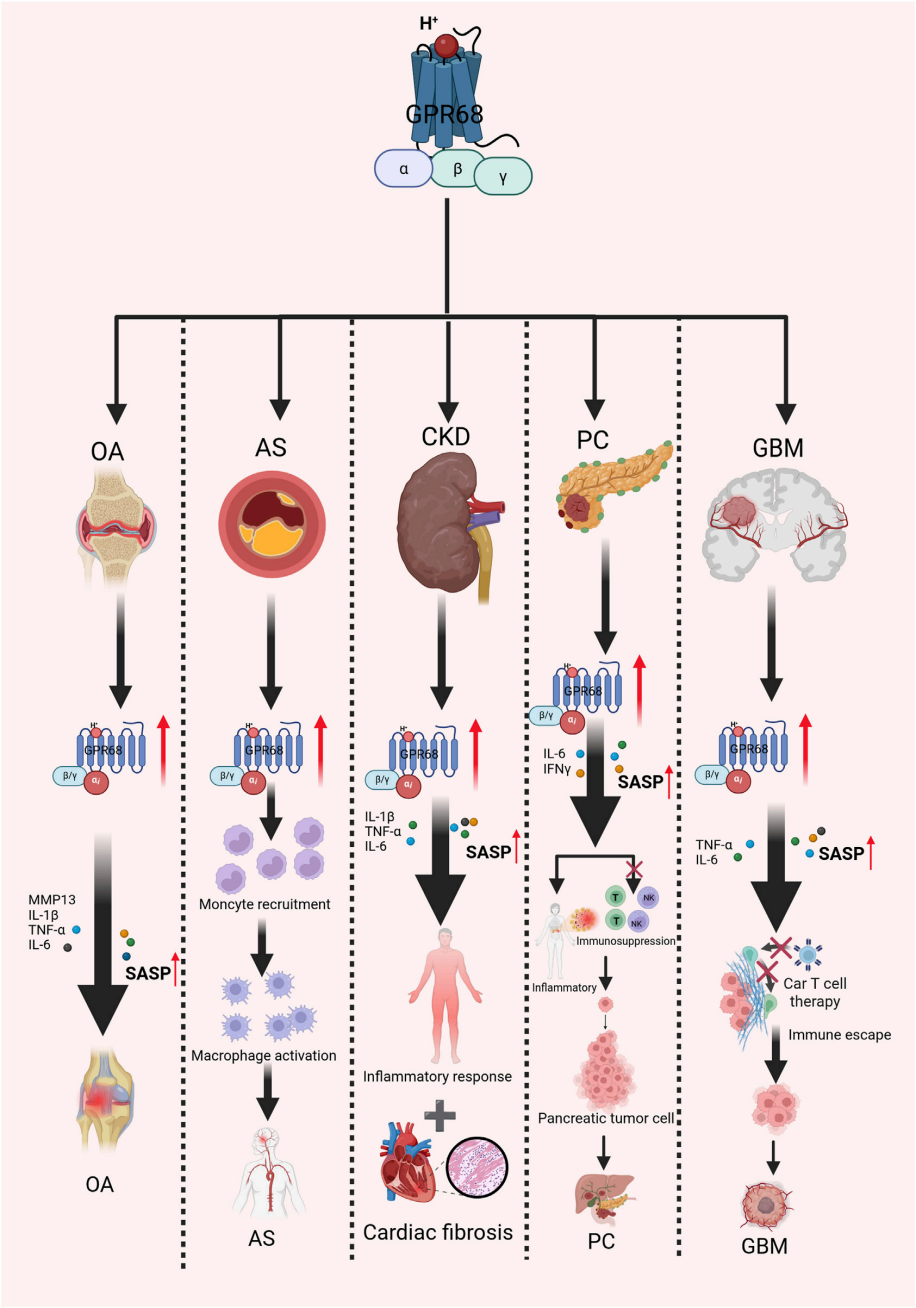
Mechanism of action of GPR68 in aging-related diseases. It explores the association between GPR68 and multiple age-related diseases. Activation of GPR68 releases multiple inflammatory mediators, intensifying inflammatory responses and thereby accelerating the progression of several age-related diseases to a certain extent. Abbreviations: AS, atherosclerosis; CKD, chronic kidney disease; GBM, glioblastoma; IFN-γ, interferon-γ; NK, natural killer; OA, osteoarthritis; SASP, senescence-associated secretory phenotype; T, T lymphocyte cell; TNF-α, tumor necrosis factor alpha.

In recent years, the relationship between aging and disease progression has gradually been clarified, and factors such as inflammation and extracellular acidification are considered to be crucial to the aging process (Arnett, 2003; Jha et al., 2024; Zhang et al., 2024). As a pH-sensing G protein-coupled receptor (pH-GPCR), GPR68 serves a crucial function in sensing acidic changes in the extracellular environment and regulating inflammatory responses (Matsingos et al., 2024; Justus et al., 2024; Wiley et al., 2018; Merchant and Ling, 2023). The pH-GPCR family was first described in 2003 by Ludwig et al. and consists of GPR4, GPR65 and GPR68 (Ludwig et al., 2003; Justus et al., 2013; Foord et al., 2005). These receptors belong to the GPCR family. GPCRs are seven-transmembrane receptors and the largest family of cell signaling receptors. GPCRs are encoded by more than 800 genes in the human genome, accounting for about 3% of the human genes, responding to a variety of cell signals, and regulating multiple physiological processes (Vassilatis et al., 2003).GPR68 function links it to the pathogenesis of multiple age-related diseases, highlighting its potential as a therapeutic target (Rowe et al., 2021). To more clearly illustrate the relationship between GPR68 and age-related diseases, we provide descriptions through both charts and text, as shown in Figures 1, 2.

Changes in extracellular proton concentration have an important impact on the cells physiological and pathological processes. Because the GPR68 receptor acts as a proton sensor, it can respond to changes in extracellular proton concentration and regulate various biological functions by stimulating the Gq/11–PLC–IP_3_/Ca^2+^ axis (Yu et al., 2024). Research indicates that GPR68 is significantly associated with the progression of multiple diseases, particularly in maintaining homeostasis and functional integrity of hematopoietic stem cells during aging (Wiley et al., 2018; He et al., 2025). Aging accelerates the secretion of inflammatory factors (including cytokines and chemokines), especially IL-1, IL-6, and TNF-α. These cytokines are known as senescence-associated secretory phenotype (SASP) factors, and during aging, their release induces long-term persistent low-grade chronic inflammation (Maciel-Barón et al., 2016). Aging-related diseases (e.g., cardiovascular diseases, diabetes, and neurodegenerative diseases) are often accompanied by local acidification in the affected tissues. GPR68 can sense changes in extracellular pH and may be involved in the regulation of inflammatory responses through signal transduction pathways (de Vallière et al., 2022). To more clearly describe how GPR68 influences disease progression by regulating cytokines, we have organized the results into a summary table, as shown in Table 1. Endothelial dysfunction is an important feature of the inflammatory response, and it is associated with a variety of aging-related diseases, such as heart failure, cardiovascular disease, and diabetes (Zuchi et al., 2020; Xu et al., 2021). Research suggests that GPR68 contributes to endothelial function regulation, with investigations demonstrating that GPR68 activation is related to an increase in endothelial cell permeability and inflammatory response, and GPR68 inhibitors may help protect the endothelial barrier function (Tomura et al., 2005).

**TABLE 1:** GPR68 regulates the progression of multiple diseases through cytokines.

Data source	Diseases	Inflammatory factor	Pathogenesis	Findings	References
Human study	Osteoarthritis	IL-1β/TNF-α	NF-κB signaling pathway	Activation of GPR68 exacerbates the inflammatory response and cartilage degradation	Khan et al. (2023)
Human study	Bronchial asthma	CTGF/IL-6	GPR68/Gq/phospholipase C (PLC)/IP_3_/Ca^2+^ signaling pathway	Extracellular acidification may induce airway remodeling by upregulating CTGF and IL-6 expression	Matsuzaki et al. (2011)
Animal study	Inflammatory bowel disease (IBD)	TNF/hypoxia	NF-κB signaling pathway	Under acidic conditions, TNF and hypoxia-induced GPR68 expression is enhanced, resulting in positive feed-forward regulation of GPR68 activity and supporting a role for GPR68 in the pathogenesis of IBD	de Vallière et al. (2016)
Animal study	Glioblastoma multiforme	ATF4	Ferroptosis signaling pathway	Specific inhibition of GPR68 can induce glioblastoma death through an iron death signaling pathway while preserving healthy nerve tissue	Williams et al. (2024)
Human study	Pancreatic ductal adenocarcinoma	IL-6	cAMP/PKA/cAMP signaling pathway	GPR68 is significantly upregulated in cancer-associated fibroblasts (CAFs), and its activation enhances IL-6 expression through the cAMP/PKA/CREB signaling pathway, promoting the development of PC	Wiley et al. (2018)
Animal study	Colonic inflammation	IL-10	A significant enrichment of IFN-γ in T lymphocytes and IL-23 in T helper cells in the colon	GPR68 expression promotes intestinal injury and induces colitis	Perren et al. (2023)
Human study	Vascular disease	NA	GPR68 deficiency in arterial endothelial cells results in acute vasodilation and chronic impaired outward vascular remodeling	GPR68 is an essential flow sensor in the arterial endothelium and is a key signaling component in cardiovascular pathophysiology	Xu et al. (2018)
Animal study	Melanoma	NA	GPR68 inhibits IFN-γ expression in tumor-infiltrating CD8 T cells and NK cells and the expression of inflammatory cytokines in mouse spleen	GPR68 inhibits lymphocyte infiltration and promotes melanoma growth	Ye et al. (2023)
Human study	PC	IL-6	GPR68 increases IL-6 expression and secretion in cancer-associated fibroblasts	GPR68 expression stimulates fibrosis and inflammatory signaling, promotes nodal fiber proliferation and ischemic necrosis, and mediates poor prognosis in patients with PC	Cornwell et al. (2023)
Human study	Cutaneous neurofibromas	NA	GPR68 activates cAMP and Ras/MAPK signaling	GPR68 promotes the growth of cutaneous neurofibromas through cAMP and Ras/MAPK signaling	Mazuelas et al. (2024)
Human study	Hepatocellular carcinoma	IL-6, IL-8, and CCL-2	GPR68 in cancer-associated fibers promotes the release of IL-6, IL-8, and CCL-2	GPR68 supports hepatocellular carcinoma progression	Yamanaka et al. (2021)

Abbreviations: ATF4, activating transcription factor 4; CAFs, cancer-associated fibroblasts; cAMP, cyclic adenosine monophosphate; CTGF, connective tissue growth factor; CREB, cAMP, response element-binding protein; CCL-2, chemokine (C-C Motif) ligand 2; IFN-γ, interferon-γ; PC, pancreatic cancer; IL-10, Interleukin-10; IP_3_, inositol 1,4,5-trisphosphate; IBD, inflammatory bowel disease; NA, not applicable; NF-κB, nuclear factor kappa-light-chain-enhancer of activated B cells; NK, natural killer; PLC, phospholipase C; TNF-α, tumor necrosis factor-alpha.

**FIGURE 3:**
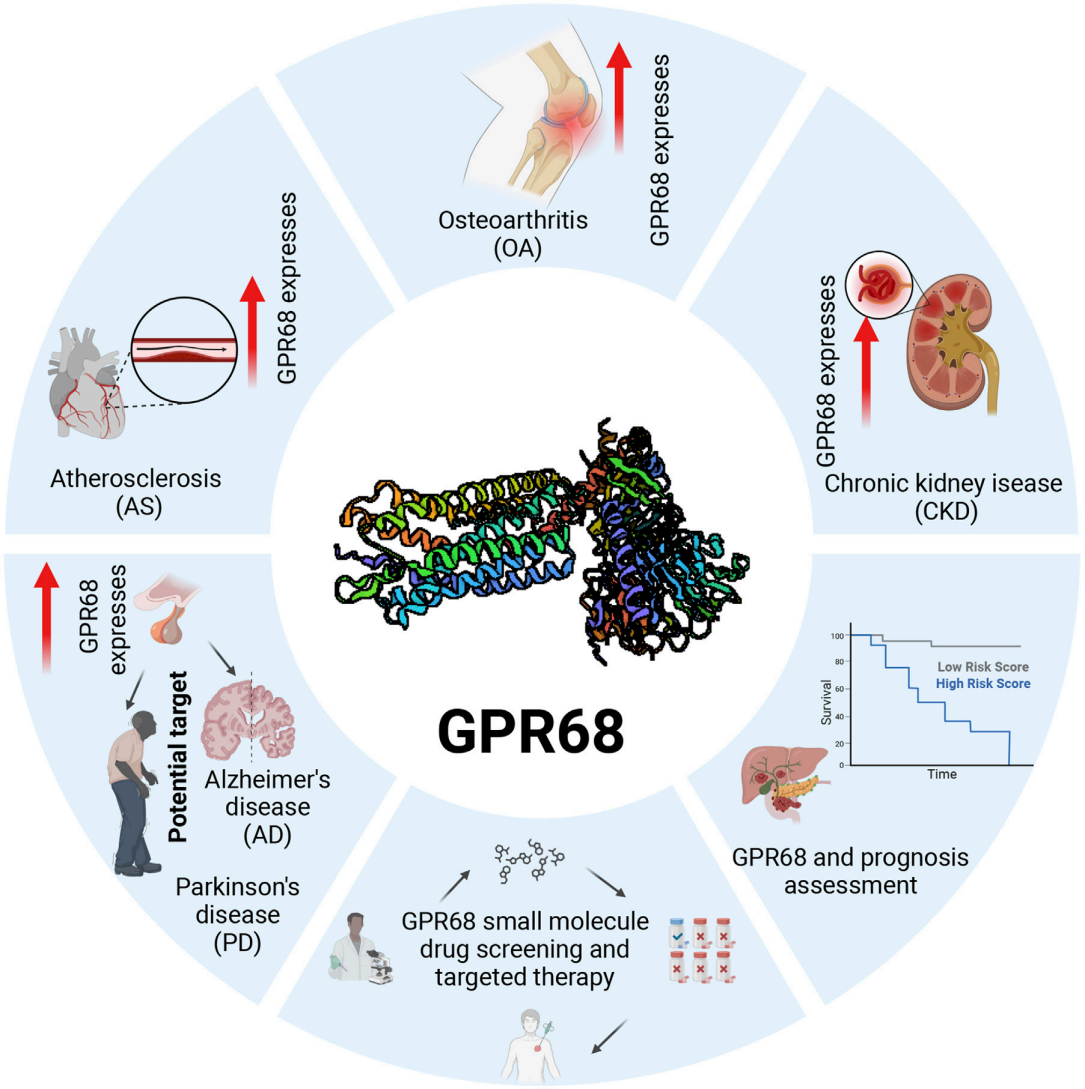
Expression and clinical application potential of GPR68 inaging-related diseases. GPR68 is upregulated in multiple age-related diseases, indicating its potential value as a therapeutic target. Screening for specific small-molecule drugs targeting GPR68 holds promise for treating various age-related conditions. Prospective evaluation of treatment regimens for age-related diseases can validate the specific therapeutic effects of GPR68-targeting small-molecule drugs. Abbreviations: AS, atherosclerosis; CKD, chronic kidney disease; GPR68, G protein-coupled receptor 68; OA, osteoarthritis.

The relationship between GPR68 and inflammation is complex, encompassing diverse cell types and signaling cascades. This review describes the structure and function of GPR68 in detail, exploring the mechanism by which GPR68 regulates the development of aging-related diseases and analyzing the clinical application potential of GPR68 in disease treatment and prognosis assessment, with the goal of promoting the development of novel therapeutic strategies based on GPR68 receptors. We illustrate the therapeutic potential of GPR68 in age-related diseases in Figure 3.

## 2 The proton-sensing receptor—GPR68

GPR68 primarily activates downstream signaling pathways by sensing extracellular acidity changes, thereby participating in the regulation of physiological and pathological processes such as inflammation, cell proliferation, and apoptosis.

### 2.1 The discovery and development of GPR68

GPR68, alternatively referred to as ovarian cancer G protein-coupled receptor 1, was initially identified in a human ovarian cancer cell line HEY. As research continued, the scientific community gradually adopted the name GPR68 to more accurately reflect its identity as a member of the GPCR family (He et al., 2025; Imenez Silva and Wagner, 2022). Associated research has demonstrated that the GPR68 gene resides on chromosome 14 and possesses three experimentally validated transcript variants; these variants code for identical GPR68 protein, with variations existing solely in the 5′untranslated region. The distinctions could affect the mRNA expression levels, subcellular localization, and response to specific signals. GPR68 has an open reading frame of 1095 nucleotides and can encode a protein of 365 amino acids (Xu and Casey, 1996).

In recent years, some researchers have classified GPR68 as a pH-GPCR whose activation depends on the extracellular pH value (Imenez Silva and Wagner, 2022). The activation state of GPR68 is measured by inositol phosphate formation (Imenez Silva and Wagner, 2022). The discovery of GPR68 was made in the context of intensive research into a family of GPCRs that respond to a variety of ligands, including light, odor, taste, hormones, and neurotransmitters (Vassilatis et al., 2003; Hauser et al., 2017). As molecular biology and genomics techniques continue to advance, GPCRs are becoming increasingly important in research on disease progression, and GPR68 is one of them.

### 2.2 The structure of GPR68

**FIGURE 4:**
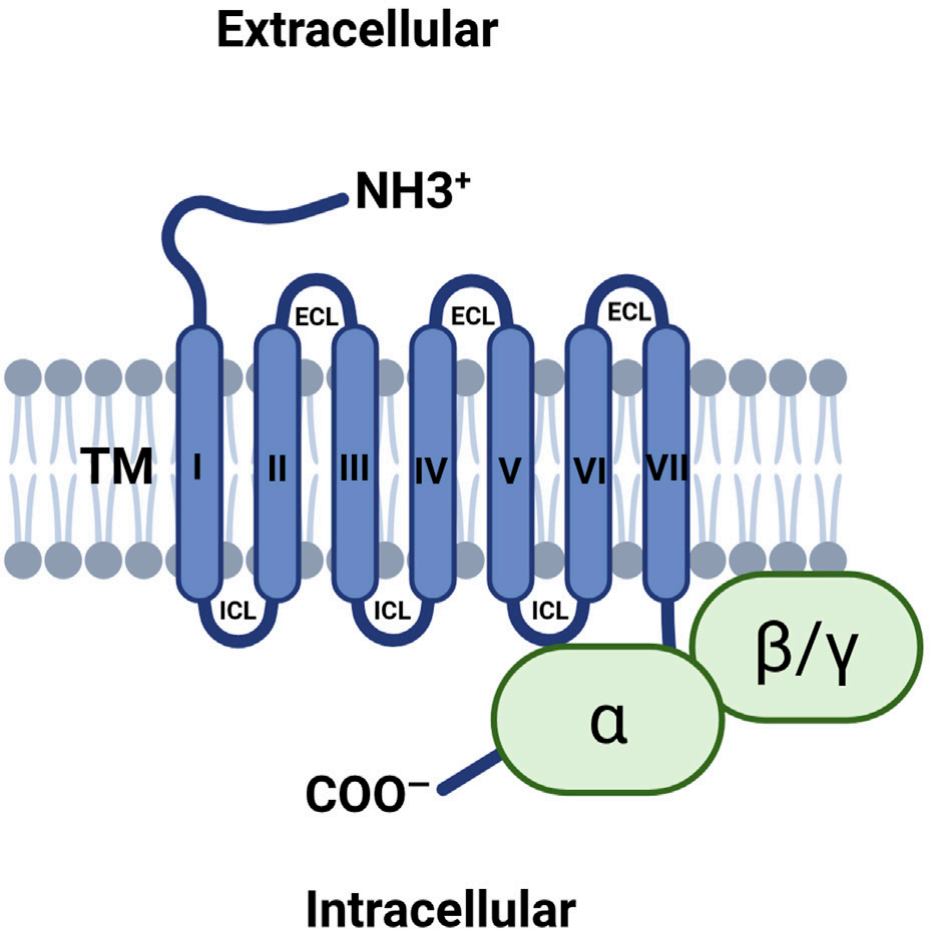
Illustrating the transmembrane structure of GPR68. GPR68 includes an extracellular N-terminal, an intracellular C-terminal, and seven transmembrane structures with three extracellular and three intracellular loops. Abbreviations: ECL, extracellular loop; ICL, intracellular loop; TM, transmembrane.

GPR68 is a member of the GPCR superfamily, which includes an extracellular N-terminal, an intracellular C-terminal, and seven transmembrane structures with three extracellular and three intracellular loops, as shown in Figure 4 (Sisignano et al., 2021). The N terminal of GPR68 lies outside the cell and contains histidine residues that are critical for proton sensing. In the unprotonated state, histidine residues interact with each other through hydrogen bonding to maintain the deactivated state of the receptor. After protonation, these residues may lose their hydrogen bonds, resulting in a conformational change of the receptor. The C-terminal of GPR68 is located inside the cell and is involved in interaction with G proteins, as well as receptor regulation and signal transduction, and the C-terminal may also be involved in the receptor internalization and degradation process.

As a unique GPCR, the crystal or cryo-electron microscope structure of GPR68 has not yet been determined, in stark contrast to the situation with many other GPCRs. As early as 2003, a three-dimensional (3D) prediction model for GPR68 was proposed (Ludwig et al., 2003), and in the past decade, different methods have been used to analyze the structure of GPR68. In 2021, the SWISS-MODEL server was used to analyze the 3D structure of the GPR68 protein, and AlphaFold modeling was used to observe the 3D structure of the generated GPR68 protein. However, the structure of GPR68 has not been fully analyzed (Jumper et al., 2021; Studer et al., 2021). Although studies have reported that GPR68 may affect the development of aging by regulating the acidic microenvironment, the relevant regulatory mechanisms have not been well elucidated, which may be attributed to the fact that the structure of GPR68 has not been thoroughly studied (Chandra et al., 2016). Therefore, it is of great significance to strengthen studies on its structure and function in the future, specifically in relation to the activation of GPR68 in acidic microenvironments. For example, the relevant researchers inserted a ring-arranged fluorescent protein into the third intracellular ring of GPR68 to engineer a genetically encoded fluorescence reporter gene activated by GPR68, called “iGlow.” (Ozkan et al., 2021).

### 2.3 GPR68 expression levels

The levels of GPR68 expression in different tissues of the normal human body are different. For example, it is expressed in the heart, spleen, lung, kidney, small intestine, brain, testis, and placenta, but not in the skeletal muscles, liver, thymus, pancreas, prostate, ovary, and colon (Wiley et al., 2019). For some specific cell types, GPR68 is expressed in peripheral blood leukocytes (Xu and Casey, 1996) (including T cells (McAleer et al., 2018) and neutrophils (Murata et al., 2009)), thyroid cells (Afrasiabi et al., 2006), liver cells (Matsingos et al., 2024), aortic smooth muscle cells (Liu et al., 2010), and airway smooth muscle cells (Ichimonji et al., 2010; Saxena et al., 2012). In addition, GPR68 is most richly expressed in the pituitary gland, followed by the esophageal mucosa, cerebellum, and lung (Wiley et al., 2019). The mechanism by which GPR68 exerts its effects depends not only on expression levels but primarily through changes in extracellular acidity and tension stimuli. We cannot judge its function based solely on expression levels; instead, we must rely on specific environmental triggers. High expression merely indicates that functional responses will be more rapid and agile under specific triggering conditions. The reason for the differential expression of GPR68 in different tissues may be that it performs unique functions in different tissues.

Moreover, GPR68 exhibits notable elevation in multiple aging-related diseases, such as cancer, osteoarthritis (OA), and others. In pancreatic cancer (PC), it was found that the average expression level of GPR68 in 147 patients with PC was 10.5 times higher than that in 165 healthy individuals (Wiley et al., 2018). Through clinical data analysis, GPR68 was found to be highly expressed in head-and-neck squamous cell carcinoma and to correlate with disease progression and patient survival (Zhang et al., 2020). Therefore, GPR68 may be a potential therapeutic target and prognostic marker for the management of head and neck squamous cell carcinoma. In studies on OA, GPR68 was found to be expressed in human cartilage and was associated with the degradation of the extracellular matrix during OA development. Studies have also shown that the high expression of GPR68 is linked to the severity of extracellular matrix degeneration and OA progression; thus, enhanced GPR68 expression may aggravate the progression of OA and lead to serious dysfunction (Khan et al., 2023).

## 3 Pathogenic mechanisms involving GPR68

Inflammatory sites exhibit localized acidification due to heightened metabolic activity or tissue damage, leading to decreased extracellular pH. H^+^ (hydrogen ion) directly binds to the N-terminal domain of GPR68, inducing conformational changes that activate Gq/11 proteins. This activation subsequently modulates inflammatory responses through multiple pathways—including Ca^2+^ signaling channels, the MAPK/ERK pathway, and ion channels—thereby influencing disease progression under specific conditions.

### 3.1 GPR68 and the inflammatory response

Studies indicate that activation of the GPR68 receptor facilitates inflammatory signaling, particularly in intestinal inflammation and age-related chronic inflammation (Ichimonji et al., 2010; Khan et al., 2023; Perren et al., 2023; Yoshida et al., 2024; Matsuzaki et al., 2011; Hutter et al., 2018). In a colitis experiment on mice, the GPR68 small molecule inhibitor ogremorphin (OGM) was used to diminish pro-inflammatory cytokine levels, including TNF and IL-6, and fibrotic factor TGF-β1, and reduce the degree of colon inflammation in mice, suggesting that GPR68 may regulate the inflammatory response through the release of regulatory factors/inflammatory factors (de Vallière et al., 2021).

GPR68 promotes certain aging-related pathological processes under specific conditions. For example, in chronic kidney disease (CKD), the increased expression of GPR68 on human monocytes promotes the release of TNF-α, IL-6, and other pro-inflammatory cytokines, which aggravate systemic inflammation and fibrosis (Sarakpi et al., 2023). TNF-α and IL-6 demonstrate strong connections with numerous inflammatory conditions, encompassing inflammatory bowel disease (IBD), rheumatoid arthritis, multiple sclerosis and atherosclerosis (AS) (Sarakpi et al., 2023; Gonzalez Caldito, 2023; Tanaka et al., 2014; Hirano, 2021). The above studies suggest that the role of GPR68 in chronic inflammation in aging-related diseases may be achieved by regulating the SASP. The composition of the SASP is complex and dynamic, including a variety of pro-inflammatory factors, immune regulatory factors, cell growth factors, and matrix proteases (Xiong et al., 2024). These factors work together to affect the behavior of surrounding cells, including inducing the senescence of surrounding cells, and participate in the chronic inflammatory response of tissues. The expression level of the SASP usually increases with aging, and it exhibits strong associations with the development of multiple age-related disorders, encompassing cardiovascular diseases (Ferroni et al., 2003), diabetes (Pradhan et al., 2001), and cancer (Cuollo et al., 2020; Ortiz-Montero et al., 2017; Faget et al., 2019).

Relevant studies have shown that the removal of senescent cells can reduce the level of pro-inflammatory cytokines in the SASP *in vivo*, thereby reducing aging-related inflammation and tissue damage (Birch and Gil, 2020). During the aging process, GPR68 promotes the expression and secretion of pro-inflammatory factors like TNF-α and IL-8, resulting in a persistent chronic inflammatory response in the body and a variety of aging-related diseases (Wiley et al., 2018; Chandra et al., 2016; Tylutka et al., 2024; Pan et al., 2023; Lin et al., 2023; Bruunsgaard et al., 2000).

Given the role of GPR68 in chronic inflammation, investigating its modulatory impact on inflammatory processes holds substantial medical importance, as it could advance research into age-associated disorders and facilitate the identification of innovative therapeutic targets.

### 3.2 GPR68 and the immune response

The expression of GPR68 may affect immune cell function, including macrophages, neutrophils, dendritic cells, and T cells, and thereby affect the inflammatory response (Justus et al., 2024). Relevant studies have shown that macrophages and granulocytes are GPR68 positive in immunohistochemical clinical samples of human duodenum, indicating that GPR68 is expressed in macrophages, granulocytes, and other immune cells (de Vallière et al., 2022). In a clinical study of IBD, it was discovered that GPR68 mRNA expression levels within the affected mucosal tissue of IBD patients exhibited notably elevated values than those observed in non-IBD controls. *In situ* hybridization experiments on non-inflammatory and inflammatory tissues of patients with IBD and mucosal samples of non-IBD subjects found that compared with non-inflammatory tissues, the expression of GPR68 in the macrophages of inflamed tissues was increased (de Vallière et al., 2022). Related studies have shown that GPR68 is involved in the inflammatory response mediated by dendritic cells (Aoki et al., 2013) and macrophages (Okajima, 2013).

Tumor studies have shown that GPR68 may regulate immune cells through cytokines/inflammatory factors and thus affect tumor progression and initiation. For instance, in a mouse melanoma model, GPR68 knockout was noted to elevate the number of effector CD8^+^ T cells, induce T cell proliferation and migration enhancement, and increase the synthesis of cytokines interferon-γ (IFN-γ), TNF-α, and granzyme B, ultimately reducing the degree of melanoma invasion (Cao et al., 2021). Activation of GPR68 may inhibit the activity of some immune cells, thus providing good conditions for the immune escape of tumor cells and the occurrence and development of tumors. For example, the collection of melanoma tissue from wild-type mice and GPR68-knockout mice and analysis of tissue infiltration by immune cells, such as lymphocytes, monocytes, neutrophils, and natural killer (NK) cells, revealed a low accumulation of CD8^+^ T cells and NK cells in wild-type mouse melanoma tissues, while a high accumulation of CD8^+^ T cells and NK cells was observed in GPR68-knockout mouse melanoma tissues. This indicates that GPR68 expression inhibited the infiltration of melanoma tissues by CD8^+^ T cells and NK cells (Ye et al., 2023). GPR68 is expressed in cancer-associated fibroblasts (CAFs) and immune cells and serves a pivotal function in a variety of diseases by regulating cytokine production. These cells release a variety of cytokines into the tumor microenvironment that affect the function of nearby immune cells. For example, in pancreatic and colorectal cancers, CAFs express GPR68, which increases the secretion of proinflammatory cytokines, facilitates inflammatory signaling, and affects the tumor immune response (Wiley et al., 2019).

### 3.3 The GPR68 signaling pathway associated with aging progression

The regulatory effect of GPR68 can be realized through a variety of intracellular signaling pathways, including Gq/11–PLCβ–IP_3_/Ca^2+^, adenylate cyclase (AC)/cAMP/PKA/cAMP response element-binding protein (CREB). When human pancreatic cells are exposed to an acidic microenvironment, GPR68 activates the NF-κB signaling pathway and promotes the secretion of the pro-inflammatory factor IL-8, suggesting that GPR68 plays a catalytic role in the inflammatory response of human pancreatic cells (Chandra et al., 2016). IL-8 is a strong and important pro-inflammatory factor during aging that may cause tissue damage and functional decline (Tylutka et al., 2024; Lin et al., 2023). Therefore, the correlation between GPR68 and the NF-κB signaling pathway is related to the inflammatory response, and GPR68 may affect the aging process through the inflammatory factors TNF-α and IL-8, thereby affecting the occurrence and development of aging-related diseases. The AC/cAMP signaling pathway is also one of the important signaling pathways of GPR68. GPR68 activates AC through the Gs protein to increase the concentration of cAMP. cAMP activates PKA, which regulates several downstream effector molecules and transcription factors. The AC/cAMP signaling pathway serves a pivotal function in a variety of physiological and pathological processes, especially in inflammatory responses during cell proliferation and aging (Zeng et al., 2020).

These pathways are not only involved in cell proliferation, survival, and inflammatory response, but also closely related to the occurrence and development of a variety of aging-related diseases. Overall, the potential of GPR68 as a drug target for the treatment of aging-related diseases is gradually being recognized, but drug development targeting GPR68 faces great challenges. Future studies are needed to further elucidate the specific mechanisms of action of GPR68 in different aging-related diseases and how to effectively modulate its activity to treat the disease.

## 4 Effects on aging-related diseases

Given the important role of GPR68 in inflammation and immunity, focusing on the effects of GPR68 on aging-related diseases and their mechanisms is critical.

### 4.1 Osteoarthritis

OA is a chronic inflammatory aging-related disease characterized by joint inflammation, cartilage loss, and joint pain (Jeon et al., 2018). More than 80% of people over 65 years of age have OA (Wieland et al., 2005). To some extent, GPR68 modulates the progression of OA. Experimental studies on mouse OA models found that the expression of GPR68 is positively correlated with matrix metallopeptidase 13 levels, indicating that the activation of GPR68 could affect the development of OA chondrocytes through catabolism and matrix degradation, thereby contributing to disease progression (Khan et al., 2023). GPR68 may serve a function in the development of OA through the following signaling pathways: the NF-κB signaling pathway and the Ras homolog family member A/Rho-associated coiled-coil containing protein kinase pathway. Furthermore, activation of GPR68 may promote the activation of NF-κB, leading to the release of pro-inflammatory cytokines, thereby enhancing inflammatory signal transduction and cartilage degradation (Khan et al., 2023; Tang et al., 2022). GPR68 influences the development of OA by regulating the expression of multiple cytokines, such as TNF-α and IL-1β (Sok et al., 2013; Akeson and Malemud, 2017). In one study, researchers studied the effects of GPR68 in a mouse model of OA. The results showed that mice lacking GPR68 had lower levels of T cell activation and markedly reduced arthritis symptoms. This suggests that the presence of GPR68 is closely related to the proliferation and activation of T cells, further confirming the importance of GPR68 in the degree of inflammation and immune response in OA.

### 4.2 Atherosclerosis

Vascular aging refers to the structural and functional changes that occur in the vascular system due to aging. Dysfunction of the vascular system can lead to various aging-related diseases such as giant cell arteritis and AS (Guo et al., 2022). In the pathogenesis of AS, GPR68 may be involved in the occurrence and development of the disease by affecting the function of vascular endothelial cells and the inflammatory response. In the early stage of AS, the aggregation of monocytes and macrophages is a typical feature. Activation of GPR68 can promote the migration of monocytes to the inflammatory site, stimulate the activation of macrophages, and increase the inflammatory response. Studies have shown that GPR68 is essential for the physiological function of blood vessels, especially in sensing hemodynamic changes (Xu et al., 2018). Changes in hemodynamics can induce endothelial cells to release pro-inflammatory cytokines, further aggravate the local inflammatory response, promote intra-arterial lipid deposition, and exacerbate AS (Ma et al., 2024). The role of GPR68 in regulating cellular response to acidosis, as well as the acidic characteristics of the atherosclerotic plaque microenvironment (Xiao et al., 2023), suggests that GPR68 may serve an important function in the pathogenesis of AS.

### 4.3 Chronic kidney disease

CKD is a common inflammatory disease in the elderly. It is a long-term condition of reduced kidney function that usually manifests as a decreased estimated glomerular filtration rate (eGFR) or the presence of protein in the urine. With the increasing aging of the global population, the incidence of CKD has increased markedly in the elderly population, especially in adults over 65 years of age, with almost 40% affected (Merchant and Ling, 2023). Relevant studies have shown that the expression of GPR68 in CKD tissues is markedly increased, suggesting that GPR68 may be involved in the regulation of the pathophysiological process of CKD. It has been reported that CKD upregulates GPR68 expression in monocytes and enhances the secretion of pro-inflammatory cytokines (TNF-α, IL-1β, and IL-6), thereby promoting inflammatory signaling and, under certain conditions, amplifying systemic inflammation and cardiac fibrosis (Sarakpi et al., 2023; Yoshida et al., 2021). The effect of GPR68 on CKD may be mediated by the Gq and Gs signaling pathways, through which GPR68 activates the conversion of PI to diacylglycerol and IP_3_, resulting in an increase in intracellular calcium ion concentration. This increase in calcium concentration enhances the expression of pro-inflammatory cytokines, which contribute to the inflammatory response in CKD (Guo et al., 2022).

### 4.4 Alzheimer’s disease

Alzheimer’s Disease (AD) is a progressive neurodegenerative disorder primarily characterized by memory impairment, cognitive decline, and behavioral changes. AD is the most common type of dementia among the elderly, accounting for approximately 60%–70% of all dementia cases (Dolphin et al., 2024). GPR68 may play a regulatory role in AD. Although no direct evidence currently exists to prove GPR68’s involvement in AD, researchers have noted its expression in mammalian brains and speculate that it may participate in the pathological processes of AD (Khan and He, 2017). GPR68 and GPR4 both belong to the pH–GPCR family and are classified as Class A orphan receptors, exhibiting numerous similarities. Through the integration of transcriptomic association analysis and weighted gene co-expression network analysis, GPR4 was identified as one of the predictive biomarkers for AD, providing statistical evidence for its significant association with AD risk (Zhou et al., 2023). Although no direct therapeutic studies targeting GPR68 in AD have been reported to date, existing research on GPR4 and its predictive value in AD provides a rationale. Future work will therefore use additional technical approaches to explore the potential of GPR68 as a predictive biomarker for AD.

### 4.5 Parkinson’s disease

Parkinson’s disease (PD) is a chronic, progressive neurological disorder that primarily affects motor function. Its pathological features include degeneration of dopaminergic neurons in the substantia nigra and the formation of Lewy bodies (Koeglsperger et al., 2023). The typical symptoms of PD include tremor, muscle rigidity, bradykinesia, and postural instability (Harrison et al., 2025). Additionally, non-motor symptoms such as cognitive impairment, depression, hyposmia, and sleep disorders are also commonly observed in PD patients (Chahine et al., 2025; Chen, 2018; Sohail et al., 2017). GPR68 may exert a regulatory effect on PD. Although no direct evidence currently exists to prove GPR68’s role in PD, researchers have proposed that GPR68 may participate in the pathological processes of PD (Khan and He, 2017). GPR68 shares numerous similarities with GPR4, as both belong to the pH–GPCR family and are classified as Class A orphan receptors. Findings from GPR4 research may provide important insights for future studies targeting GPR68 in PD. Researchers discovered that at the cellular level, activation of GPR4 is closely associated with neuronal apoptosis. Knockout of GPR4 significantly reduces neuronal apoptosis (Haque et al., 2020). Research has found that the GPR4 inhibitor NE52-QQ57 significantly reduces neuronal damage and improves motor function and spatial memory in mouse PD models (Haque et al., 2021). Although no direct experimental studies targeting GPR68 in PD have been reported to date, research on GPR4 in this disease suggests that GPR68 is likely to emerge as a potential therapeutic target for PD in the future.

### 4.6 Glioblastoma

Glioblastoma (GBM) is one of the most common primary malignant brain tumors of the adult central nervous system. GBM poses a serious health challenge to patients due to the proliferation of tumor cells and their invasion of normal tissues, which usually results in a poor prognosis and survival times of less than 1 year. Recent studies have shown that the activation of GPR68 under acidic conditions activates the endoplasmic reticulum stress activating transcription factor 4 (ATF4) signaling pathway, which is a novel survival pathway in glioblastoma. The activation of the GPR68-ATF4 signaling pathway can promote the production of the pro-inflammatory factors IL-6 and TNF-α and helps glioblastoma cells to evade immune surveillance, thus enhancing the growth and survival of glioblastoma cells (Kumari et al., 2021; Li et al., 2024). GPR68 is expressed in macrophages, and the activation of GPR68 may lead to the polarization of macrophages into the M2 phenotype, which usually has immunosuppressive properties and contributes to tumor growth and metastasis (Justus et al., 2024). One study used OGM, a novel small molecule inhibitor of GPR68, to explore the role of GPR68 in GBM cells and found that blocking the GPR68 signaling pathway led to ferroptosis of tumor cells in GBM cell lines. These findings suggest that GPR68 may be a new target for the treatment of GBM (Williams et al., 2024).

### 4.7 Pancreatic cancer

PC is a malignant tumor characterized by aggressive growth, local invasion, and distant metastasis. A study has shown that the expression of GPR68 in PC is markedly higher than that in most normal pancreatic cells (Wiley et al., 2019). This high expression may be closely related to tumor development, possibly due to the involvement of GPR68 in the interaction between tumor cells and the tumor microenvironment. In PC tissues, the GPR68 expression levels correlate with tumor aggressiveness and prognosis, making it a potential biomarker and therapeutic target. In an acidic microenvironment, GPR68 activation in pancreatic CAFs upregulates IL-6 via the cAMP/PKA/CREB axis (Wiley et al., 2019). Furthermore, relevant studies have shown that GPR68 functionally inhibits the expression of IFN-γ in tumor-infiltrating CD8^+^ T cells and NK cells, which may lead to tumor immune escape and further promote tumor growth (Ye et al., 2023).

GPR68 is involved in the pathogenesis of a variety of aging-related diseases, including inflammatory diseases and cancer. By studying the relationship between GPR68 and inflammatory factors, researchers hope to develop new therapeutic strategies to target the inflammatory mechanisms of these diseases (Sarakpi et al., 2023; Elemam et al., 2022). As an important pH-GPCR, GPR68 is involved in the regulation of the inflammatory response and cytokine production. Given its central role, GPR68 is a promising therapeutic target, and future research is expected to translate its mechanism of action into novel treatments for aging-related diseases.

## 5 Clinical application potential of GPR68

Considering that GPR68 can influence the progression of multiple aging-related diseases, it has excellent potential for clinical application in early diagnosis, targeted therapy, and prognosis evaluation.

### 5.1 Early diagnosis

GPR68 plays an essential role in regulating cellular functions, especially inflammatory responses. Specifically, the activation of GPR68 can induce the release of cytokines, chemokines, and other inflammatory mediators, thereby triggering or aggravating the inflammatory response (van Setten, 2022). In chronic inflammation, the expression of GPR68 is usually markedly elevated, which is positively correlated with the severity of inflammation and the clinical score (Justus et al., 2024). GPR68 expression is markedly increased in multiple tumors and correlates with poor patient prognosis, implying that GPR68 is a potential prognostic marker for cancer. Specifically, through the analysis of tumor samples, it was found that the expression level of GPR68 in breast cancer tissues was positively correlated with the metastasis and recurrence of the tumor; therefore, GPR68 expression provides important information for the early detection and diagnosis of cancer (Elemam et al., 2022). In addition, GPR68 expression in neuroendocrine tumors shows its potential as a diagnostic marker. For instance, GPR68 expression in pheochromocytoma and cervical adenocarcinoma was shown to be positively correlated with patient survival, suggesting its value in diagnosing these cancers (Herzig et al., 2019).

### 5.2 Targeted therapy

Given its involvement in regulating inflammation, tumors, and fibrosis, inhibiting GPR68 activity is a potential therapeutic strategy for aging-related diseases.

In CKD, GPR68 has been found to promote the activation of the inflammasome by increasing the intracellular calcium ion concentration, thus aggravating the inflammatory response. Interestingly, the GPR68 inhibitor OGM suppresses this inflammatory response, reducing tissue damage and improving the prognosis of chronic diseases. GPR68 is closely associated with tumor proliferation, invasion, and metastasis. In clinical studies, the inhibition of GPR68 expression and blocking of GPR68 signaling slowed the growth and spread of tumor cells. For example, in GBM, a novel small molecule drug, OGM, inhibits GPR68 by activating ATF4, which induces ferroptosis and inhibits tumor progression (Williams et al., 2024). Similarly, GPR68 facilitates tumor escape from immune surveillance, and knockout of GPR68 in melanoma and prostate cancer inhibits tumor proliferation (Yan et al., 2014; Li et al., 2009) Chronic inflammation is one of the primary drivers of fibrosis. Certa Therapeutics is investigating the GPR68 antagonist FT011 for the treatment of renal fibrosis (Cornell et al., 2024). FT011 has shown significant efficacy in multiple fibrosis-related disease models; in diabetic cardiomyopathy and myocardial infarction models, it markedly reduced cardiac fibrosis and improved function (Zhang et al., 2013; Zhang et al., 2012). GPR68 expression is significantly elevated in the inflamed mucosa of patients with IBD, particularly in those with Crohn’s disease and ulcerative colitis, where its expression levels correlate positively with disease severity (de Vallière et al., 2022). Research indicates that treatment with the GPR68 inhibitor OGM in acute and chronic mouse colitis models reduces mucosal inflammatory responses, providing the first evidence that targeting GPR68 ameliorates mouse colitis (de Vallière et al., 2021). GPR68 also plays an important regulatory role in the hematopoietic system. Researchers using hematopoietic cell-specific GPR68 conditional knockout mice have shown that targeting GPR68 enhances the function of aging hematopoietic stem cells, providing a novel therapeutic strategy for age-related hematopoietic decline (He et al., 2025).

In summary, GPR68 is a potential therapeutic target for inflammatory diseases, cancer, fibrosis, and age-related decline in hematopoietic function. Targeting GPR68 could provide new therapeutic options for patients who do not respond well to conventional therapies. To better evaluate the therapeutic efficacy of targeting GPR68 across age-related diseases, we have compiled the relevant results in Table 2. To gain a deeper understanding of the mechanism of action of GPR68, we systematically compiled information on GPR68 agonists and their biological function, as shown in Table 3.

**TABLE 2:** Inhibitors of GPR68 and their therapeutic roles in various diseases.

Data source	Compound	Compound functions	Cell type/model	Findings	References
Human study	GPR68-I	Inhibitor	Fibroblasts and CD14^+^ human monocytes	The inhibition of GPR68 alleviates acute and chronic DSS (glucan sulfate)-induced colitis in mice	de Vallière et al. (2022)
Animal study	ogremorphin (OGM)	Inhibitor	Glioblastoma cells	GPR68 plays a pro-survival role in glioblastoma through the GPR68-ATF4 signaling pathway, and its inhibitor OGM can induce ferroptosis	Williams et al. (2024)
Animal study	GPR68-I	Inhibitor	DSS-induced acute and chronic colitis models	The inhibition of GPR68 increases the expression of the genes associated with intestinal inflammation and immune response	de Vallière et al. (2021)
Animal study	Homoharringtonine	Inhibitor	Chronic kidney disease (CKD) mouse model	GPR68 promotes cardiac inflammation and fibrosis, and inhibiting the function of GPR68 can improve the cardiac inflammation and fibrosis caused by CKD, thereby improving the survival rate	Yoshida et al. (2024)
Animal study	Asengeprast (FT011)	Antagonist	Neonatal cardiac fibroblasts an experimental rat model of MI-induced LV heart failure	FT011 attenuates cardiac remodeling and systolic dysfunction following experimental myocardial infarction	Zhang et al. (2013)
Animal study	Asengeprast (FT011)	Antagonist	A rat model of diabetic nephropathy	Reflecting the ability of FT011 to inhibit the action of locally-active growth factors involved in fibrosis, FT011 also attenuates cardiacremodeling and dysfunction in experimental diabetic cardiomyopathy	Williams et al. (2013)
Animal study	Asengeprast (FT011)	Antagonist	HEK293T, MV-4-11, etc., the mouse MV-4-11 xenograft model	FT001 displayed potent biochemical and cellular activity, translating to excellent *in vivo* activity in a mouse xenograft model (MV-4-11)	Millan et al. (2017)
Animal study	Clock-mutant mice carrying a deletion of exon 19 of the Clock gene (Clock)	pathway inhibition	circadian loss-of-function mutant mouse model	Genetic disruption of circadian function alone indirectly suppresses GPR68 and is sufficient to significantly attenuate CKD-associated cardiac inflammation and fibrosis, providing genetic proof-of-concept for therapeutic targeting of GPR68	Yoshida et al. (2021)
Animal study	GRP68 knockout	Genetic depletion	Mouse model of melanoma	GPR68 knockout reduced melanoma growth, suggesting that GPR68 deficiency in the host cells impedes tumor growth	Li et al. (2009)
Animal study	GRP68 knockout	Genetic depletion	Colitis mouse model	Genetic deletion of GPR68 has been found to lead to reduced disease severity concerning intestinal inflammation in a colitis mouse model	Perren et al. (2023)
Animal study	GRP68 knockout	Genetic depletion	Mouse model of cerebral ischemia	GPR68 knockout mice exhibit more severe neurological deficits in a cerebral ischemia model, suggesting a neuroprotective function	Wang et al. (2020)

Abbreviations: CKD, chronic kidney disease; CART, cocaine and amphetamine-regulated transcript; cAMP, cyclic adenosine monophosphate; DSS, dextran sulfate sodium; HEK293T, human embryonic kidney 293T cells; NCF, neonatal cardiac fibroblasts; TGF-β, transforming growth factor-beta.

**TABLE 3:** Activators of GPR68 and their biological modulation.

Data source	Compound	Compound functions	Cell type/model	Findings	References
Animal study	Ogerin	Agonist	HEK293T cells expressing GPR68	Ogerin acts as a regulator of GPR68, playing a role in regulating fear memory, affecting TGF-β signaling, and possibly other GPR68-related brain functions	Huang et al. (2015)
Human study	Ogerin	Agonist	Primary human lung fibroblasts	Ogerin inhibits TGF-β-induced myofibroblast differentiation of fibroblasts from multiple organ systems	Bell et al. (2022)
Human study	Ogerin	Agonist	Human cutaneous neurofibroma-derived Schwann cells	The activator of GPR68 (Ogerin), in combination with the MEK inhibitor (MEKi) Selumetinib, was able to reduce the viability of primary Schwann cells derived from human skin neurofibromatosis and induce the differentiation and death of these cells	Mazuelas et al. (2024)
Human study	MS48107	Agonist	HEK293T cells expressing GloSensor cAMP	The allosteric activity of GPR68 was greatly increased, demonstrating high selectivity, bioavailability, and brain permeability in mice	Mazuelas et al. (2024)
Human study	Lorazepam	Agonist	HEK293T cells expressing GPR68	It mobilizes calcium to generate and activate cAMP signals	Yu et al. (2019)
Human study	139 (CART (42-89)_9-28_)	Agonist	Flp-In T-REx 293 cells	Activation of the Gq/11 pathway mediated calcium mobilization, and the Gs pathway mediated cAMP signaling	Foster et al. (2019)
Human study	Rat 148 (Corticotropin_17-40_)	Agonist	Flp-In T-REx 293 cells	Activation of the Gq/11 pathway mediated calcium mobilization, and the Gs pathway mediated cAMP signaling	Foster et al. (2019)
Human study	128 (Osteocrin_33-55_)	Agonist	Flp-In T-REx 293 cells	Activation of the Gq/11 pathway mediated calcium mobilization, and the Gs pathway mediated cAMP signaling	Foster et al. (2019)
Human study	Sulazepam	Agonist	HEK293T cells expressing GPR68	Selective activation of cAMP signal	Yu et al. (2019)
Animal study	Ogerin	Agonist	GPR68 (−/−) Mouse fear-conditioning model	Suppresses recall in fear conditioning	Huang et al. (2015)
Animal study	Sulazepam	Agonist	Allergic asthma mouse model	Reduced airway resistance	Nayak et al. (2021)
Animal study	Isx (3,5-disubstituted isoxazoles)	Agonist	Mouse ischemic myocardial infarction model	Promotes the expression of myogenesis and survival promotion genes in myocardial cells in subepicardial tissue after myocardial infarction	Russell et al. (2012)

Abbreviations: CART (42-89)_9-28_, cocaine- and amphetamine-regulated transcript (42-89)_9-28_; Corticotropin_17-40_, adrenocorticotropic hormone_17-40_; Gq/11, G protein alpha-q/11; Gs, G protein alpha-s; HEK293T, human embryonic kidney 293T cells; Isx, 3,5-disubstituted isoxazoles; MEK, mitogen-activated protein kinase Kinase; MEKi, MEK, inhibitor; Osteocrin_33-55_, Osteocrin Fragment_33-55_; TGF-β, transforming growth factor-beta.

### 5.3 Prognostic evaluation

GPR68 contributes to the evaluation of the prognosis of patients with chronic inflammation and tumors. In one study, it was found that GPR68 mRNA expression at the site of inflammation in the colon was markedly elevated in patients with IBD, and this elevation was positively correlated with disease severity and the clinical prognostic score (Justus et al., 2024). Moreover, activation of GPR68 promoted inflammation and fibrosis of the heart, which suggests that GPR68 is a potential player in the occurrence and progression of heart disease, and its high expression may be associated with poor prognosis (Yoshida et al., 2024). In oncology, several studies have shown a close relationship between GPR68 and tumor prognosis, drug resistance, and tumor metastasis. By assessing GPR68 expression in a patient’s tumor, it is possible to more accurately predict patient survival and tumor development. For example, in patients with breast cancer, high GPR68 expression is associated with higher recurrence and mortality rates, which confirms the prognostic potential of GPR68 and provides a basis for individualized treatment plans for patients (Elemam et al., 2022). Notably, in oral cancer studies, GPR68 deletion can aggravate chemically induced oral dysplasia, indicating that GPR68 activation may have a protective effect in oral cancer (Shore et al., 2023). The role of GPR68 may vary across different tumors, potentially inhibiting tumor growth or promoting its progression. Potential reasons include tissue-specific cellular characteristics and differences in the tumor microenvironment. As a potential therapeutic target, GPR68 should not be approached with a uniform treatment strategy across all diseases. We must thoroughly investigate its specific functions in different pathological states to fully unlock its potential as a therapeutic agent.

GPR68 is not only a promising tumor biomarker but also a potential therapeutic target for chronic inflammatory and neoplastic diseases, and it can be used to evaluate the prognosis of patients and select appropriate therapeutic strategies. Future studies should focus on revealing the specific mechanisms underlying the effects of GPR68 and its role in chronic inflammation and different types of cancer to develop more effective treatment strategies for improving patient survival and quality of life.

## 6 Conclusions and future perspectives

Currently, research on GPR68 has gradually increased and revealed the important function of GPR68 in regulating aging-related diseases. However, there are still many challenges that limit a more comprehensive understanding of GPR68. Here we provide some highly representative, but not exhaustive, examples of the challenges facing research on GPR68.

### 6.1 From structure to mechanism: deepening our understanding of GPR68

The structure of GPR68 is crucial; without a high-resolution structure, the lack of *in vivo* causality between GPR68 activation and downstream pathological outcomes remains unresolved. Although the crystal or cryo-electron microscopic structures of many GPCRs have been determined, the exact structure of GPR68 requires further elucidation due to the absence of key experimental data. Advances in technology have opened up the possibility of utilizing multiple approaches to study the specific structure of GPR68. For example, in molecular dynamics simulation, a three-dimensional model of GPR68 has been proposed based on the homology modeling of other known GPCRs. Using the C-X-C chemokine receptor 4 architecture as a template, researchers first generated a 3D model of GPR68. Subsequently, they produced a set of 2,900 additional models through elastic network extension. In total, a repository of 3,307 models has been established, laying the foundation for further research on the specific structure of GPR68 (Rowe et al., 2021).

In the future, the structure of GPR68 can be studied by X-ray crystallography, nuclear magnetic resonance, and the accuracy of the structure of GPR68 can be improved by combining various experimental techniques. Importantly, combining structural studies of GPR68 with computer prediction and validation through virtual screening and mutation analysis is a powerful strategy for elucidating the structural function of GPR68. For example, computer-aided drug design was used to screen possible GPR68 ligands and predict GPR68 binding patterns. Site-specific mutation of key amino acid residues of GPR68 was performed, and the effect of mutation on protein activity was analyzed by functional experiments (Basak, 2013). These research strategies will help to further understand the structure and function of GPR68 and promote the application of GPR68 in the biomedical field.

Considering the effects of GPR68 on aspects of immune cell function, the inflammatory microenvironment, and the tumor immune microenvironment, future studies should focus on the multiple roles of GPR68 in mechanism, immunity, and signal transduction. For instance, we focus on how to regulate the GPR68 status change when the microenvironment pH changes. In a weakly alkaline microenvironment, GPR68 is stabilized in a deactivated state by hydrogen bonding. With decreased pH and acidification of the microenvironment, GPR68 activity increases, and this response exhibits pronounced signal context-dependency, meaning that the same proton stimulus can trigger distinct G-protein coupling or β-arrestin recruitment patterns depending on cell type, receptor density, and concurrent signaling inputs. Consequently, GPR68 activation may affect immune cell function in inflammation and tumors in a context-dependent manner. Therefore, the future mechanism research can focus on the following aspects: In the tumor microenvironment, how GPR68 affects cell signaling in response to pH changes in the tumor microenvironment should be explored. Furthermore, the interaction between GPR68 and other signaling pathways is also the focus of research, because GPR68 may affect the tumor immune microenvironment by regulating the function of immune cells. Moreover, other aspects of the research are equally noteworthy, such as how GPR68 affects the proliferation, activation, and apoptosis of immune cells in inflammatory and tumor microenvironments. Additionally, how GPR68 regulates the activity of T cells by changing the acidic environment of cells should be studied. These studies could elucidate the underlying mechanisms by which GPR68 affects chronic inflammation and the immune microenvironment and provide a theoretical basis for the development of novel chronic inflammation therapies and cancer immunotherapies.

### 6.2 Toward clinical translation: challenges and strategies

Advancing the clinical use of GPR68 in inflammatory diseases and cancer represents an urgent goal. The development of small molecule drugs faces several challenges, such as poor compound selectivity, low drug efficiency, and considerable side effects. Several strategies are proposed to address these issues: First, high-throughput screening techniques are used to screen compounds that specifically bind to GPR68. Second, studying how small molecule drugs selectively target GPR68 could help in designing more effective treatments. Finally, novel drug delivery systems to improve the accuracy of small molecule drugs that target GPR68 should also be developed. In terms of biomarker identification, GPR68 may be used as an inflammatory and tumor-specific marker because GPR68 is upregulated at inflammatory sites and in multiple tumor cell lines. Future studies should focus on the identification of biomarkers for age-related diseases, which could help improve the efficiency of early diagnosis and effectively evaluate the prognosis of patients with tumors. By evaluating patient prognosis, we can provide patients with an individualized treatment plan, which would improve the accuracy and effectiveness of treatment. In terms of technological innovation, emerging biotechnology, such as clustered regularly interspaced short palindromic repeats (CRISPR) gene editing and single-cell sequencing, should be used to deeply study the function of GPR68 and its role in inflammation and tumors. In addition, early-stage clinical trials of GPR68 to evaluate its therapeutic potential in chronic inflammation and different types of cancer should be designed and performed. In clinical trials, long-term follow-up should be used to evaluate the efficacy and safety of GPR68-targeted therapy. This will help build more reliable clinical evidence.

We systematically reviewed and synthesized current knowledge regarding the challenges associated with GPR68, including specificity, off-target effects, drug delivery, and experimental data.

To address the low specificity of GPR68, we conducted an analysis focusing on the complexity of its ligand recognition and signaling mechanisms. The primary ligand for GPR68 is extracellular H^+^, with its activity dependent on changes in extracellular pH. Additionally, its ligand recognition involves specific amino acid residues, such as extracellular histidine and acidic residues, whose protonation states are crucial for receptor activation. Another significant reason for its low specificity is the cross-reactivity with other pH-GPCRs, such as GPR4 and GPR65.

Off-target effects of GPR68 small-molecule drugs primarily stem from the high structural and functional similarity among members of the GPCR family. This similarity may cause small-molecule drugs to interact with other GPCRs while binding to GPR68. Addressing the off-target effects of GPR68-targeting drugs demands a multi-pronged approach, which includes optimizing drug selectivity, evaluating mechanisms of action, developing specific modulators, integrating genetic analysis, and utilizing animal models to validate safety. These strategies not only enhance drug efficacy but also minimize side effects, providing safer treatment options for patients.

Small-molecule drugs targeting GPR68 face numerous challenges in drug delivery and efficacy. GPR68 is expressed in multiple tissues and cell types, but its expression levels and function vary significantly across different environments. This heterogeneity makes precise targeting of specific tissues or cells difficult, thereby reducing delivery efficiency. Most small-molecule drugs targeting GPR68 are prone to degradation and inactivation in the *in vivo* environment, particularly under acidic conditions. This instability limits effective accumulation at target sites, resulting in poor drug stability. Although GPR68 plays a critical role in specific barrier tissues, small-molecule drugs often struggle to penetrate these barriers, hindering drug delivery to the intended location.

The systematic analysis of GPR68 trial data requires a multi-faceted approach. First, as a pH-GPCR, it plays crucial roles in diverse physiological and pathological processes, including neuroprotection, inflammation regulation, and tumor microenvironment modulation. Experimental designs can employ techniques such as molecular dynamics simulations, gene expression analysis, Western blotting, and flow cytometry to investigate GPR68 activation mechanisms under varying pH conditions and its downstream signaling pathways. Additionally, its role in inflammatory bowel disease warrants attention, as its expression correlates with disease activity and its inhibition reduces colonic inflammation. The synthesis of experimental data should integrate *in vivo* and *in vitro* models, including mouse models, cell cultures, and clinical sample analyses, to comprehensively evaluate its function and potential therapeutic value. Integrating multidimensional data can provide deeper insights into the physiological and pathological mechanisms of GPR68.

### 6.3 Concretization of future work: a comprehensive research case study of GPR68 in atherosclerosis

To thoroughly investigate the function of GPR68 and its mechanisms in inflammation and tumorigenesis, we present a detailed overview across four key areas: CRISPR knockout application, *in vivo* validation of inhibitors, high-throughput screening, and clinical trial design. We chose AS as an example to show the relationship between GPR68 and AS. GPR68 knockout mice can be generated via CRISPR-Cas9. An AS model will be established by feeding mice a high-fat diet for over 16 weeks. The formation of atherosclerotic plaques will be compared between GPR68 knockout mice, wild-type mice, and wild-type mice treated with the GPR68 inhibitor OGM. Serum levels of inflammatory mediators (IL-6, TNF-α) will be measured by ELISA to validate the effects of GPR68 knockout and pharmacological inhibition on atherosclerotic progression. High-throughput screening of multiple GPR68 small-molecule inhibitors will be conducted using a HEK293 cell line stably expressing GPR68 and the calcium indicator GCaMP6s as the screening platform. The Enamine compound library will be selected for screening, with automated screening and virtual screening assisting in the efficient batch screening of GPR68 small-molecule inhibitors. Clinical evidence supporting GPR68 as an AS biomarker remains insufficient. A prospective cohort study and biomarker validation will be conducted, enrolling 300 AS patients and 200 healthy controls. Blood samples will be collected to measure GPR68 expression in blood cells and serum levels of the inflammatory cytokine IL-6. Patients underwent follow-up for up to 3 years. Subsequently, the relationship between GPR68 expression and AS progression will be assessed.

Continuous efforts in the above aspects will broaden the research and application prospects of GPR68. Future research will not only help reveal its potential in the treatment of inflammation and cancer but may also advance the development of treatment options for other age-related diseases associated with GPR68, fully realizing its potential as a therapeutic target.

## Abbreviations

AC, adenylate cyclase; AS, atherosclerosis; ATF4, activating transcription factor 4; 3D, three-dimensional; CAFs, cancer-associated fibroblasts; CKD, chronic kidney disease; CREB, cAMP response element-binding protein; CRISPR, clustered regularly interspaced short palindromic repeats; GBM, glioblastoma; IBD, inflammatory bowel disease; IFN-γ, interferon-γ; NK, natural killer; OA, osteoarthritis; OGM, ogremorphin; PC, pancreatic cancer; pH-GPCR, pH-sensing G protein-coupled receptor; SASP, senescence-associated secretory phenotype.
